# Development and Characterization of a New Endoscopic Drug-Eluting Platform With Proven Efficacy in Acute and Chronic Experimental Colitis

**DOI:** 10.3389/fmed.2020.00415

**Published:** 2020-08-20

**Authors:** Ignacio Bon, Mary Cano-Sarabia, Napoleon de la Ossa, Ramon Bartolí, Vicente Lorenzo-Zúñiga

**Affiliations:** ^1^Health Research Institute Germans Trias i Pujol (IGTP), Barcelona, Spain; ^2^Catalan Institute of Nanoscience and Nanotechnology (ICN2), CSIC and The Barcelona Institute of Science and Technology, Campus de la UAB, Bellaterra, Spain; ^3^Servicio de Anatomia Patológica, Hospital Universitari General de Catalunya—Grupo Quirón Salud, Barcelona, Spain; ^4^Centro de Investigación Biomédica en Red de Enfermedades Hepáticas y Digestivas (CIBEREHD), Barcelona, Spain; ^5^Endoscopy Unit, University Hospital La Fe, Valencia, Spain

**Keywords:** drug-eluting, experimental colitis, endoscopic shielding technique, hydrogel, endoscopy

## Abstract

**Background and Aims:** Mucosal lesions refractory to biological treatments represent unmet needs in patients with inflammatory bowel disease (IBD) that require new treatment modalities. We developed and characterized a new endoscopic drug-eluting hydrogel (CoverGel) with proven efficacy in acute and chronic experimental colitis (EC) in rats.

**Methods:** CoverGel was developed based on appropriate rheological, drug release, gelation, structural, and degradation property capacities to allow endoscopic application. Experimental colitis (EC) was induced by TNBS application in rats. In acute EC 40, rats were randomized in five groups (eight each): Sham, Control, CoverGel, CoverGel + Infliximab (IFX) and CoverGel + Vedolizumab (VDZ). In chronic EC, 12 rats were randomized in two groups (six each): IFX s.c. and CoverGel + IFX. Endoscopic, histological, and blood test were performed during follow-up to evaluate clinical success. Antibodies to IFX (ATIs) were evaluated in chronic EC animal study.

**Results:** CoverGel is a biocompatible and bioadhesive reverse thermosensitive gelation hydrogel with a macroporous structure and drug release capacity. In acute EC animals treated with CoverGel + IFX or CoverGel + VDZ showed significantly clinical success (weight recovery, mucosal restoration, and bacterial translocation) as compared with controls and animals without a bioactive drug. In a chronic EC animal study, clinical efficacy was comparable in both groups. Levels of ATIs were significantly lower in animals treated with CoverGel + IFX vs. IFX s.c. (0.90 ± 0.06 μg/mL-c vs. 1.97 ± 0.66 μg/mL-c, *p* = 0.0025).

**Conclusions:** CoverGel is an endoscopic vehicle to locally deliver biological drugs with proven efficacy in acute and chronic EC in rats and induce less immunogenicity reaction.

## Introduction

Interventional inflammatory bowel disease (IBD) endoscopy has an expanding role in the era of biologic therapy. A significant number of patients have mucosal lesions refractory to biological treatments despite the availability and growing use of anti-tumor necrosis factor (TNF), anti-integrin, anti-interleukin, and new small-molecule (1, 2). These drugs have changed the way of treating IBD patients, but those situations represent unmet needs that require new treatment modalities to prolong the intervention-free period. An endoscopic shielding technique with hydrogels has been proposed by our group as a less invasive approach to reduce or postpone the need for surgical resection (3, 4).

Hydrogels or hydrophilic gels are cross-linked polymeric three-dimensional networks which present soft tissue-like elastic, non-toxic, and biodegradable properties while being responsive to stimuli (5–8). Nowadays we do not have any valid endoscopic treatment to IBD mucosal lesions in patients with refractory or loss of response to biological therapy. The purpose of the present study was to develop and characterize a new endoscopic drug-eluting platform and test its efficacy in acute and chronic experimental colitis in rats.

## Materials and Methods

### Characterization of the Drug-Eluting Platform

We have developed a new hydrogel (CoverGel) comprising specific amounts of hyaluronic acid (HA), methylcellulose (MC), and Poloxamer Pluronic-F127 (F127). This platform has been patented (WO 2018/019881 A1). The composition of CoverGel is based on the combination of different substances to create this solution, which can be prepared before the procedure.

Our goal was to improve the beneficial effects of HA onto the colonic mucosa by adding different substances to increase biocompatibility, bioadhesive, and bioactive properties and make it suitable to be administered through the endoscope's working channel. For this, we evaluated different aspects: rheological properties (viscosity, adherence, and microstructure), gelation, degradation, drug absorbance, drug release, and biocompatibility. Four different original combinations (colon dressing, CD) of prototypes of materials (CD1, CD2, CD3, CD4) were tested for viscosity, adhesion force, and microstructure. The best composition for the drug-eluting platform was different percentages of HA, MC, and PF127. All compounds of the product are well-known and have proven safety. The composition of the invention is biodegradable, is easy to administrate, remains adhered to the mucosal lesion, and is a drug-release composition.

**Rheological test:** (i) Viscosity was evaluated in a rheometer (Haake RheoStress; Thermo Fisher Scientific, Waltham, MA, USA) with a C60/1° Ti probe and a gap set of 0.053 mm. The rotation ramp went from 0–300 s^−1^ in 30 s. For each study, Viscosity (η) was measured as a function of shear rate (γ) at 22°C (room temperature) and 37°C (body temperature), (ii) Adhesion was measured with a Texture Analyzer TA.XT Plus (Stable Micro Systems, Surrey, UK). A 40-mm (diameter) disk was compressed into the gel and redrawn. The velocity of the insertion assay was 1 mm/s, and the withdrawn distance was 9 mm. Adhesion was determined at 20 and 37°C, and the (iii) microstructure of the hydrogel was assessed with scanning electron microscopy (SEM).

**Gelation assay** was carried out to determine the behavior of the hydrogel under UV light radiation at different temperatures. The following scenarios were tested: (i) 1 mL of hydrogel and 15 μL of photo-initiator solution were mixed and placed on a petri dish. The hydrogel was heated at 37°C for 30 min, and gelation was tested. Next, the hydrogel was cooled; (ii) 1 mL of hydrogel and 15 μL of photo-initiator solution were mixed and placed on a petri dish. The dish was placed under ultraviolet light 2,000 mV/cm^2^ for 3 min (AnalytikJeta UVP Crosslinker), keeping the temperature below 20°C. The hydrogel was heated at 37°C for 30 min, and gelation was tested. Next, the hydrogel was cooled; (iii) 1 mL of hydrogel was placed on a petri dish. The hydrogel was heated at 37°C for 30 min, and gelation was tested. Next, the hydrogel was cooled; (iv) 1 mL of hydrogel was placed on a petri dish. The dish was placed under ultraviolet light 2,000 mV/cm^2^ for 3 min, keeping the temperature below 20°C. The hydrogel was heated at 37°C for 30 min, and gelation was tested. Next, the hydrogel was cooled. For hydrogel observation after the irreversible episode, they were lyophilized at 50°C for 96 h and were examined by Field-Emission Scanning Electron Microscopy (FE-SEM) on a FEI Quanta 650 microscope using aluminum tape as support.

**Degradation assay** was performed to understand the degradation rate of our hydrogel under different conditions that would try to mimic the different conditions found in the gastrointestinal tract; 1 mL of hydrogel was irreversibly gelified and then submerged in 10 mL of one of the following solutions: PBS pH = 7, PBS pH = 3, PBS pH = 9, PBS pH = 11 and rat feces bacteria culture on Luria-Bertani (LB), and broth at a starting range of 10^5^ CFU/mL. The piece of gelified hydrogel was weighed before the submersion (Wo) and then on set date points (Wt) until complete dissolution of the hydrogel or until 1 month under the conditions was reached. At each time point, the medium was also changed when the weighing took place.

**Drug absorbance and drug release kinetics** of the hydrogel was also evaluated *in vitro* with mathematical models to study the capacity of our hydrogel to both release and absorb molecules of different molecular weight, in order to further study the capability of this hydrogel to act as a drug-eluting platform. For these experiments, we worked with two molecules: (i) Trypan blue, which has a molar mass of ~ 960 Daltons; and (ii) bovine serum albumin (BSA), which has a molecular weight of 66.5 kDa. For the Trypan blue experiments, first, a calibration curve was prepared by measuring the absorbance (λ = 450 nm) of different concentrations of Trypan blue diluted in water using a Uv-Vis spectrophotometer (Varioskan Flash, Thermo). SkanIt Software 2.4.1 was used to analyze results. Then, the curve equation was obtained: y = 6234.9x + 0.1008. *R*^2^ = 0.99349. Absorbance and release of BSA was evaluated with the Bicinchoninic acid (BCA) Protein Assay Kit (Pierce^TM^, Thermo Scientific) following manufacturer's instructions.

To incorporate substances inside the hydrogel, 0.25 mL of gelated hydrogel was submerged in 5 mL of 0.0005 mg/mL Trypan blue or 2 mg/mL BSA. Hydrogels incorporated the solution at room temperature for 48 h. To calculate the amount of substance incorporated into the matrix of the hydrogel, the following equation was used:

$$\text{Incorporating efficiency }\left(\%\right)=\frac{\text{V}1•\text{C}1-\text{V}2•\text{C}2}{\text{V}1•\text{C}1}\;\times 100$$

Where V1 is the original volume of the substance solution (mL); V2, the remaining volume of the substance solution after 48 h (mL); C1, the initial concentration of the substance (μg/mL); and C2, the remaining concentration of the substance (μg/mL).

Hydrogels were withdrawn from the solution and placed in a release medium (PBS). At specific time points, the amount of substance released into the medium was evaluated as mentioned before. The kinetics of drug release from the hydrogels was studied with three different models to asses which one fitted best with the release data from our experiments. The three different models were the following:

$$\begin{array}{l} \text{ Higuchi release model}:\text{Mt}=\;{\text{K}}_{\text{h}}{\text{t}}^{1/2}\\ \text{Zero}-\text{order release model}:\text{Mt}={\text{K}}_{0}\text{t}\\ \text{First}-\text{order release model}:\text{Mt}=1-{\text{e}}^{-{\text{K}}_{1}}\text{t} \end{array}$$

Where Mt is the fraction of drug released at each time point (t), and K_h_, K_0_, and K_1_ are the Higuchi release kinetic constant, the zero-order release kinetic constant, and the first-order release kinetic constant, respectively.

Lastly, the drug release mechanism was analyzed using the Korsmeyer-Peppas model:

$$\begin{array}{l} F=\left(\frac{Mt}{M}\right)=K_{m}t^{n} \end{array}$$

This is a semi-empirical model used when the exact mechanism is not known or in the case when more than one mechanism is involved in the drug release; where F is the fraction of drug released at time (t), Mt is the amount of drug released at time (t), M is the total amount of drug in the hydrogel, Km is a kinetic constant, and n is the release exponent, indicative of the drug release mechanism. n is estimated from linear regression of log (Mt/M) vs. log t; when determining the n exponent, only portions of the release curve ≤ 60% should be used.

**Biocompatibility assays (cytotoxicity, hemolysis, and acute toxicity)** were first performed to check the possible cytotoxicity induced by CoverGel, carrying out a co-culture of a cell line with different conditions involving our hydrogel. The cell line chosen for these experiments was Caco-2 (ATCC® HTB-37TM), a human colorectal adenocarcinoma cell line. For cytotoxicity evaluation, 3 × 104 cells/cm^2^ were cultured in 24-well cell plates under the following conditions: Normal culture medium (DMEM medium supplemented with 10% fetal bovine serum, 1% L-glutamine, and 1% penicillin/streptomycin) as a positive control; Normal culture medium supplemented with 10% dimethyl sulfoxide (DMSO) as a negative control; and Normal culture medium supplemented with 1%-2%-5%-10%-15% of hydrogel v/v. Cells were cultured at 37°C and 5% CO_2_ for 24, 48, 72, and 96 h. At specific time points, cell viability was evaluated using Resazurin Sodium Salt (Sigma-Aldrich).

Hemolysis test was done *in vitro*. Two milliliters of gelified hydrogel were placed in 20 mL of sterile saline and incubated for 72 h at 37°C. Afterward, the solution was collected and filtered with a 0.22-μm membrane; 2-mL blood samples were freshly collected from six male Sprague–Dawley rats into an anticoagulant tube and gently mixed. Blood was then diluted with 2.5 mL of saline. For the hemolysis test, 10 mL of the hydrogel extraction, distilled water, and saline were poured in 50 mL test tubes and placed at 37°C for 30 min. After that, 0.2 mL of diluted blood was added to each test tube with gentle shaking, and the solution was placed at 37°C for 1 h. The absorbance of the samples after 1 h was evaluated with a spectrophotometer (Varioskan Flash, Thermo) at 545 nm. SkanIt Software 2.4.1 was used to analyze results. Hemolysis ratio (%) was calculated with the following formula:

$$\begin{array}{l} \frac{\text{A hydrogel}-\text{A saline solution}}{\text{A distilled water}-\text{A saline solution}}\;\times \;100 \end{array}$$

The acute toxicity of CoverGel was evaluated in male Sprague–Dawley rats, 7 months of age (400–550 g). Animals were divided in three groups: two subjects and a control group. Each group contained three animals, and a total of nine animals were used. Rats from the subject groups were injected with 10 mL/kg of our hydrogel into the abdominal cavity, and rats from the control group were injected with 10 mL/kg saline into the abdominal cavity. Animals were observed daily after administration and evaluation of general conditions (activity, hair, feces, behavior, and other clinical signs), body weight, and mortality were done. Animals were euthanized at 3 and 7 days with anesthetic overdose, and major organs (heart, liver, spleen, lung, and kidney) were extracted, evaluated, and fixed in a 10% formaldehyde solution. Organs were then stained with hematoxylin/eosin for histopathological evaluation. Blood was also extracted, and hematological and biochemistry analyses were performed.

The effect of subcutaneous implantation of CoverGel was evaluated in six male Sprague–Dawley rats, 7 months of age (420–500 g). Rats maintained the subcutaneous implantation for 10 days. Under anesthesia, a 2-cm incision was done on the dorsal back of the rat, and 0.5 g of gelified hydrogel was implanted subcutaneously. The incision was then sutured, and rats were allowed to recover with food and water *ad libitum*. After 10 days, rats were euthanized, and the hydrogel was extracted and cut in two; one piece was placed in Carnoy solution (60% ethanol, 30% chloroform, and 10% glacial acetic acid) for histological evaluation and the other piece was lyophilized (Christ Loc-1 m, B. Braun) and evaluated using SEM.

### Endoscopy

All endoscopic evaluations were carried out using an Olympus Video bronchoscope EVIS EXERA II (BF-1T180)-type endoscope with an outer diameter of 6.0 mm and a working channel diameter of 3.0 mm. Room air was used for insufflation during the endoscopy. Endoscopic application of hydrogel was done by positioning the tip of the catheter over the mucosal lesions.

### Acute Experimental Colitis (EC) Animal Study

The EC animal model was induced by the rectal instillation of 0.6 mL of 3.5% TNBS solution in 50% ethanol (Day 0). The peak of colitis was established 3 days post administration (Day 3), and animals were examined to evaluate the affection and apply treatments. Forty male Sprague–Dawley rats (Harlan Laboratories Models SL, Barcelona, Spain) weighing 250–300 g, which lost at least 8% of the original weight (Day 0) and showed a circumferentially affected area at day 3, were included in this study. Animals were randomized in five groups: (i) Sham group, healthy animals with no administration of TNBS; (ii) Control Group, animals with EC treated with 1 mL of saline, through the endoscope; (iii) CoverGel group, animals with EC treated with CoverGel without active substance; (iv) CoverGel + Infliximab (IFX) group, animals with EC treated with CoverGel with IFX, 1 mg/mL; (v) CoverGel + Vedolizumab (VDZ) group, animals with EC treated with CoverGel with VDZ, 1 mg/mL. A total amount of 1.5 mL of CoverGel was applied in groups iii, iv, and v; thus, the dosage of the drug applied to groups iv and v was 1.5 mg of IFX or VDZ, respectively.

Animals were weighed and underwent colonoscopy on days 0, 3, and 7. Blood was extracted by a small cut on the tail, and serum was kept frozen for future evaluations. Ponderal evolution was studied as the variation (Δ) of original weight at day 0 for each rat. Four days after treatment (Day 7), animals were euthanatized by an anesthetic overdose, and liver and colon were evaluated. Ulcer site samples were cut and placed in formaldehyde for H/E staining and blinded histopathological evaluation. Histological study evaluated the maintenance of the intestinal architecture and the inflammatory cell infiltrate in a 0–3 scale for both characteristics and a final 0–6 scale. The scoring system (Table 1) followed was extracted from Ulrike Erben et al. (9). Liver was retrieved under sterile conditions and 0.5 g was places in 1 mL of saline and homogenized. Then, 0.1 mL of the homogenate was cultured on a LB Agar petri dish at 37°C for 24 h. The appearance of CFU was evaluated as a way to establish bacterial translocation to the liver, a marker, or a weak intestinal wall due to inflammation.

**Table 1:** Histological scoring system for the experimental colitis animal models.

**Inflammatory cell infiltrate**			**Intestinal architecture**		
**Severity**	**Extent**	**Score 1**	**Epithelial changes**	**Mucosal architecture**	**Score 2**
Mild	Mucosa	1	Focal erosions		1
Moderate	Mucosa and submucosa	2	Erosion	± Focal ulcerations	2
Marked	Transmural	3		Extended ulcerations ± granulation tissue ± pseudopolyps	3
Sum of scores 1 and 2:					0–6

### Chronic EC Animal Study

All animals included in this protocol underwent four rounds of TNBS colitis induction in the same fashion as the acute model, thus, induction was done on days 0, 7, 14, and 21; treatments were given on days 3, 10, 17, and 24, and euthanasia was performed on day 28. Twelve male Sprague–Dawley rats were included. Animals were randomized in two groups: (i) IFX s.c. group, animals with EC treated with 5 mg/kg of IFX subcutaneously; (ii) CoverGel + IFX group, animals with EC treated with CoverGel + IFX (1 mg/mL). A total amount of 1.5 mL of CoverGel was applied per animal; thus, the total amount of drug administered to group ii was 1.5 mg of IFX per animal at each day of treatment. All assessments were carried out the same way as in the acute EC study. Animals underwent endoscopic follow-up at days of induction, days of treatment, and the day of euthanasia. Furthermore, antibodies to IFX (ATIs) in serum were also evaluated. To separate ATIs from IFX as they might be found on blood, a protein G column and an acidic buffer treatment were used; 600 μL of a 1:10 dilution of serum was applied onto the protein G column (Ab Spin trap; GE Healthcare) and washed. Trapped ATIs were eluted with 400 μL of elution buffer (0.1 M Glycine-HCl, pH 2.7) which releases proteins trapped in the protein G column and, at the same time, dissociates ATI's-IFX complexes. Samples were eluted into neutralizing buffer (1M Tris-HCl pH 9) containing an IgG concentration of 20 μg/mL to prevent the reformation of immune complexes. Then, with the samples obtained from the protein G column, a 96-well plate was coated with 200 μL/well O/N at 4°C or for 1 h at 37°C. Plates were washed with washing buffer (PBS 0.1% Triton), and 200 μL/well horseradish peroxidase-conjugated Infliximab (HRP conjugation Kit, Abcam) (0.2 μg/mL IFX-HRP) was added for 30 min at room temperature, protecting the plate from direct light. The plate was further washed, and finally, a substrate solution (3,3′,5,5′-Tetramethylbenzidine/H_2_O_2_ 50% vol/vol) was added to the microplate. The enzyme reaction yields a blue product that turns yellow when stop solution (2 M sulfuric acid) is added. The intensity of the color measured is in proportion of the quantity of ATIs bound in the initial step; this intensity is measured by determining the optical density of each well using a microplate reader at 450 nm with a wavelength correction set to 540 nm using a spectrophotometer (Varioskan Flash, Thermo). SkanIt Software 2.4.1 was used to analyze results.

### Statistical Study

All values are expressed in median ± range unless otherwise stated. The comparison of groups was done using two-way ANOVA and Tukey's multiple comparison test as a *post hoc*. Release kinetics was studied using mean values of 6 different experiments and a non-linear regression fit to each different model equation. The Graphpad Prism 6.0 software was used to perform analysis.

### Animal Care

All animals were obtained from ENVIGO. All protocols were reviewed by the Animal Experimentation Ethics Committee of the Hospital Universitari Germans Trias I Pujol (registered as B9900005) and approved by the Departament d'Agricultura, Ramaderia, Pesca, Alimentació i Medi Natural of the Catalan Regional Government (protocols number 8,585 and 10,235) according to current national and European Union legislation regarding the protection of experimental animals. Rats were supervised daily in order to ensure animal welfare and euthanized, if required, by anesthetization with isoflurane. All animals had free access to water and food *ad libitum*. After any procedure carried on the animals, 200 mg/kg of paracetamol was administered intraperitoneally, and animals were monitored daily. Normal parameters of well-being such as hair health and secretions were analyzed.

## Results

### Development of the Drug-Eluting Platform

**Figure 1:**
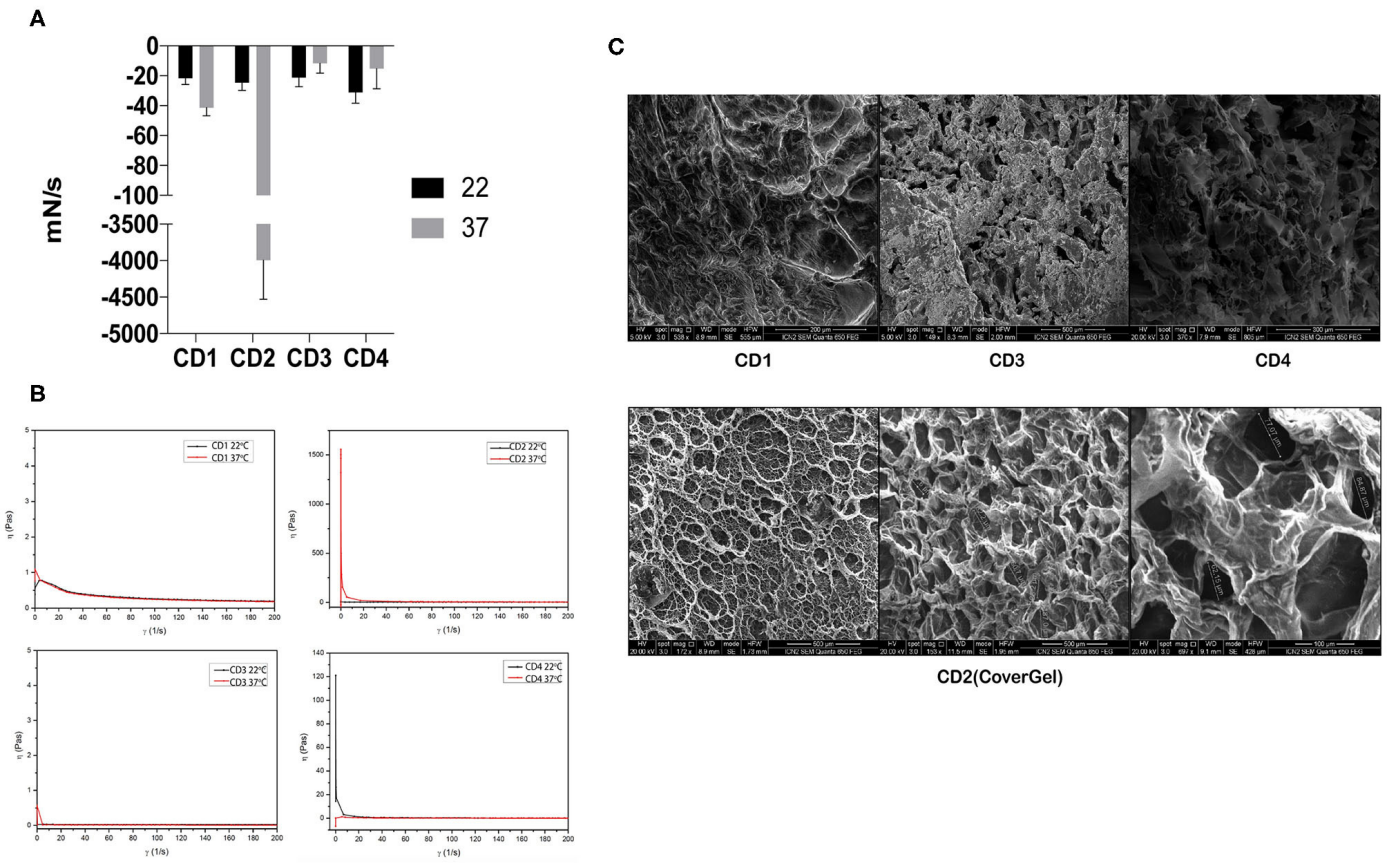
Rheological evaluations of the four original colon dressings (CDs). **(A)** Adhesion force evaluated at 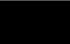 22°C and 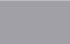 37°C expressed in mN/s. **(B)** Viscosity evaluated at 22°C (black line) and 37°C (red line) expressed in pascals. **(C)** Scanning electron microscopy images of CD1, CD3, and CD4 (top three pictures) and CD2 (bottom pictures).

All hydrogels showed a similar adhesion force at 22°C, but when temperature was raised, adhesion force of only CD2 (CoverGel) was highly increased from −25 to −3,993 mN/s (Figure 1A). Viscosity (η) of all samples was similar at 22°C, but when temperature was increased to 37°C, the viscosity of CD2 was increased from <1 Pa·s at 22°C to 1,500 Pa·s at 37°C (Figure 1B). In addition, the morphology of irreversible hydrogel was evaluated by SEM observing that it is formed by a structured network with well-defined porous distribution ranging from 50 to 300 μm (Figure 1C).

**Figure 2:**
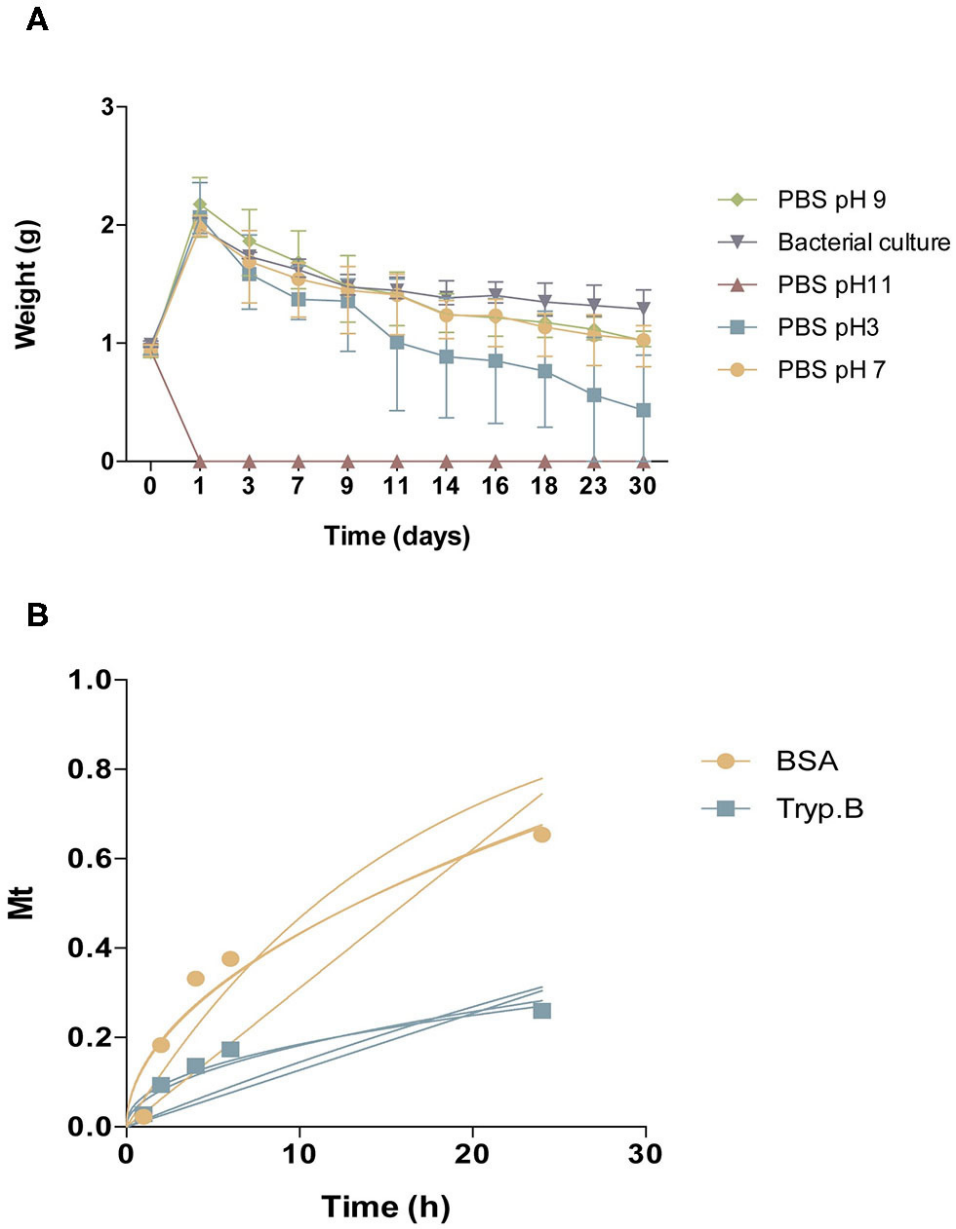
Characterization of CoverGel. **(A)** Degradation of the hydrogel when placed for 30 days under different conditions (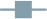 PBS pH3, 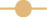 PBS pH7, 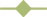 PBS pH9, 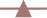 PBS pH 11, and 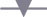 bacterial culture). Graph represents weight of hydrogel over days. **(B)** Drug-release kinetics for 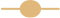 Bovine serum albumin and 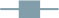 Trypan blue. Graph represents concentration of substance on release medium (Mt) over time. Lines represent the different mathematical models tested.

Reversible and irreversible gelation of the hydrogel was acquired. Hydrogel can be given smoothly through a catheter (diameter 2.0–2.2 mm) by applying a force of 370 mmHg (0.48 atmospheres). Hydrogel was also stable during 30 days at physiological-like conditions (Figure 2A). For Trypan blue, the incorporating efficacy was 58.96 ± 2.8%, and for BSA, 24.09 ± 4.34% (mean ± SD). Drug release from hydrogel was related to the concentration of the drug in the medium, which followed a Fickian diffusion. The release kinetics model that best fits our results is the Higuchi release model, being the final equation obtained for our results: for Trypan blue (Mt = 0.05760t^1/2^; *R*^2^ = 0.8986) and for BSA (Mt = 0.1369t^1/2^; *R*^2^ = 0.9167). The release exponents (n) were 0.4338 for Trypan blue and 0.5116 for BSA (Figure 2B). Table 2 shows the correlation coefficient of the different kinetic models and the Korsmeyer-Peppas mechanism model.

**Table 2:** Coefficient of determination (*R*^2^) of our data to the three kinetic release models (zero-order release model, first-order release model, and Higuchi release model) and for the drug release mechanism (Korsmeyer-Peppas model).

**Sample**	**0-order *R*^**2**^**	**1st-order *R*^**2**^**	**Higuchi *R*^**2**^**	**Korsmeyer-Peppas**
Tryp. B	0.2022	0.3361	0.8986	0.9169
BSA	0.5393	0.8324	0.9167	0.9171

**Figure 3:**
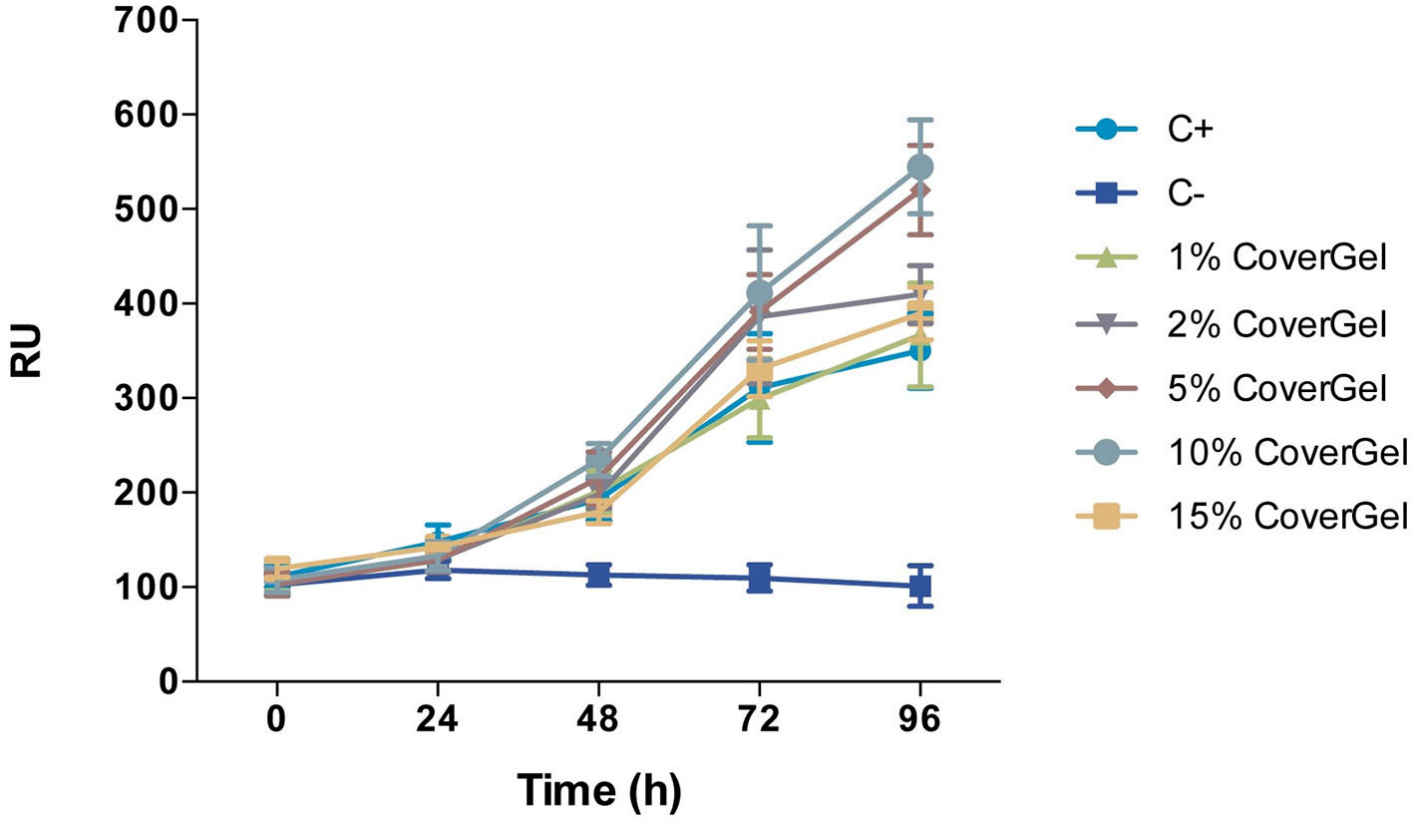
Cell viability of Caco-2 cells in conditioned medium with 1, 2, 5, 10, and 15% of CoverGel. Cells were cultured at 37°C and 5% CO_2_ for 24, 48, 72, and 96 h. Values are expressed in Relative Fluorescence Units (RFU).

CoverGel showed good biocompatibility, both in the *in vitro* and *in vivo* studies. Caco-2 cells cultured with different concentrations of our hydrogel showed increased proliferation, reaching a top at 10% when compared to the positive control of the experiment (normal culture medium) (Figure 3). Hemolysis rate caused by our hydrogel on blood from three different rats was 2.829 ± 1.135%.

**Figure 4:**
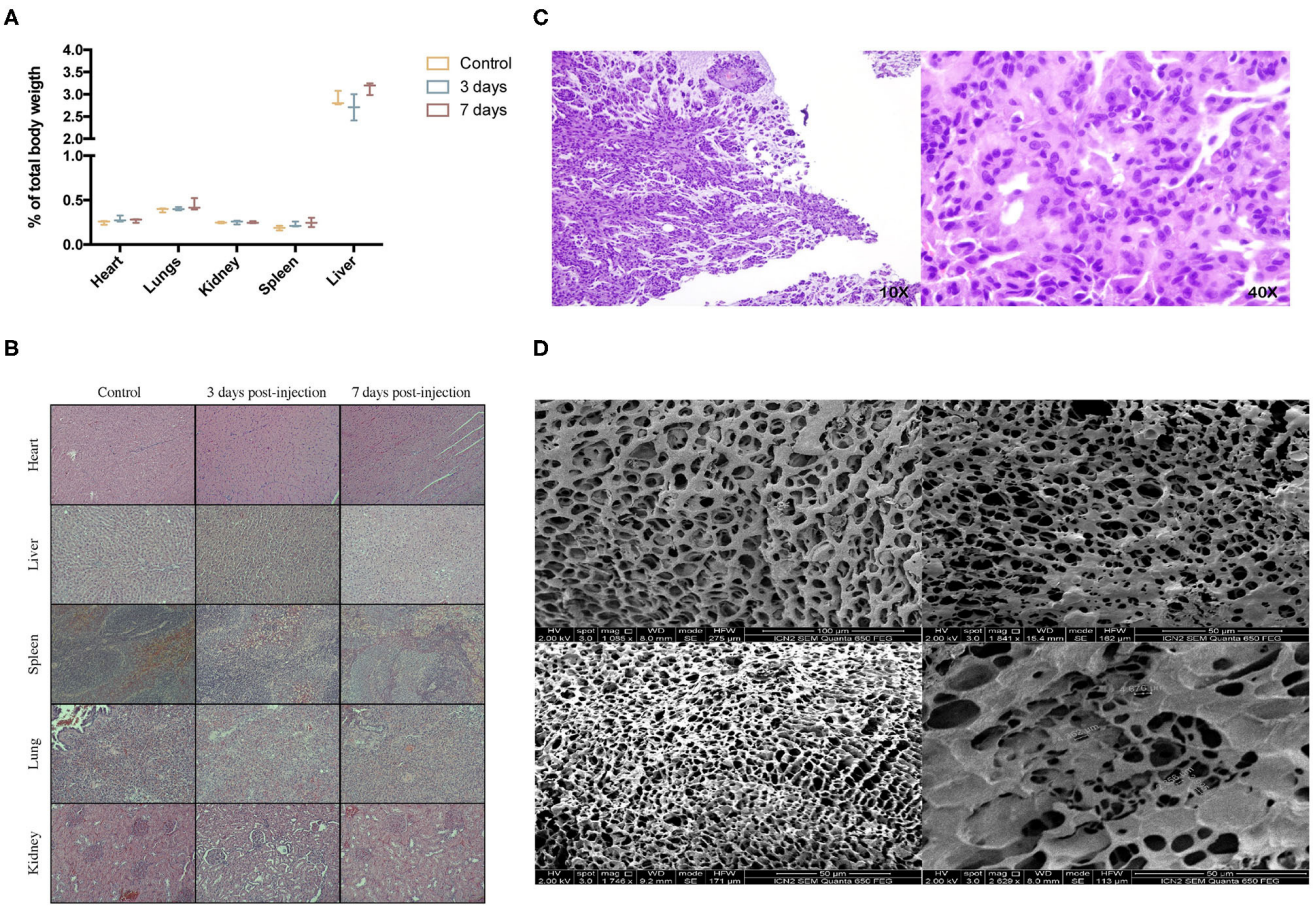
*In vivo* biocompatibility study. **(A)** % of organ weight relative to total body weight after intraperitoneal injection of 10 mL/kg of CoverGel on rats. 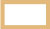 represents control rats 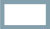 rats euthanized 3 days post injection and 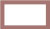 rats euthanized 7 days post injection. **(B)** Representative images of major organs H/E stainings from each group at a 40 × zoom. **(C)** H/E staining images of tissue surrounding subcutaneously placed CoverGel after 10 days post placement (10 × left image, 40 × right image). **(D)** Scanning electron microscopy images of hydrogel after 10 days post subcutaneous placement.

There was no sign of inflammation, toxicity, or tissue malformation on major organs induced by the hydrogel. Macroscopic evaluation of major organs didn't show any signs of abnormalities, and organ weight with respect to total body mass didn't present any significant differences (Figure 4A). Heart tissue didn't show signs of toxicity; cardiac myocytes maintain a good arrangement; and no hemorrhage, inflammation or necrosis is observed. In the liver, no hepatocellular degeneration occurs, and there isn't an abnormal neutrophil, lymphocyte, or macrophage infiltrate in the tissue. Tissue structure from spleen, kidneys, and lungs is maintained as well (Figure 4B). Subcutaneous placement of 0.5 g of the hydrogel showed a synovial metaplasia, a capsular surface covered by fibrohistiocytic cells that are grouped forming pseudovilli. No toxicity or inflammation was seen in the surrounding tissue, meaning that the body responded by encapsulating the hydrogel as it would do with a foreign body, but no rejection response was triggered (Figure 4C). At the time of placement, hydrogels weighed 0.5 g; when extracted, the weight was 0.95 ± 0.06 g. The platform was able to maintain its porosity and inner structure. No cell infiltration was seen 10 days after subcutaneous placement (Figure 4D).

### Efficacy of the Drug-Eluting Platform in Acute EC Animal Study

**Figure 5:**
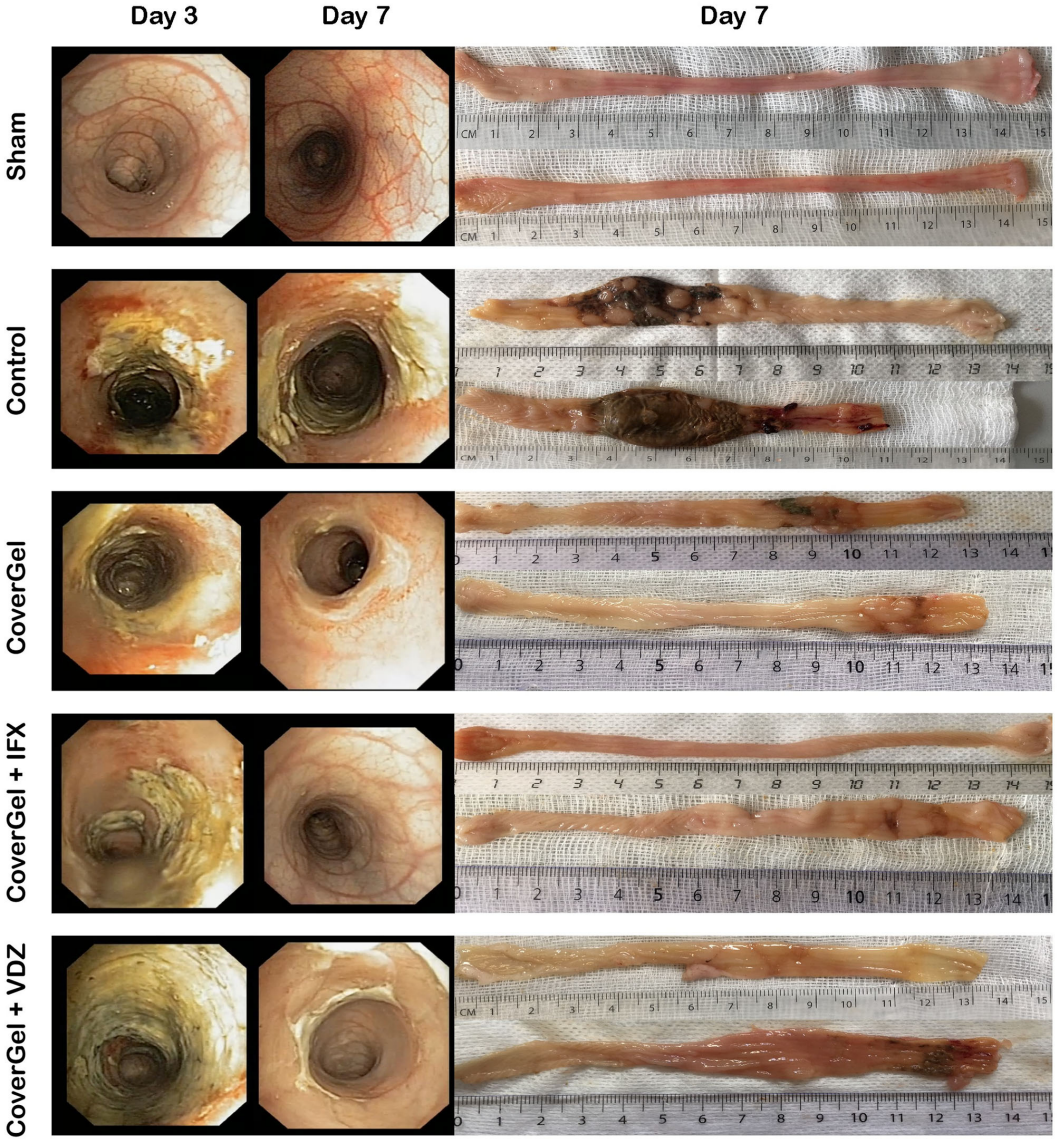
Endoscopic and macroscopic representative images of the acute EC animal model. Endoscopic images from day 3 (day of treatment) and day 7 (day of euthanasia) and macroscopic image of day 7 (day of euthanasia) from two representative animals of each group.

At day 3, all animals presented an entire circumferential affectation of the wall. Macroscopic and endoscopic evaluations at day 7 showed a complete mucosal restoration (vascular pattern, ulceration and friability) in the CoverGel + IFX and CoverGel + VDZ groups, whereas in the CoverGel group, this mucosal restoration was not complete. However, all treated groups clearly showed less mucosal damage than the Control group (Figure 5).

**Figure 6:**
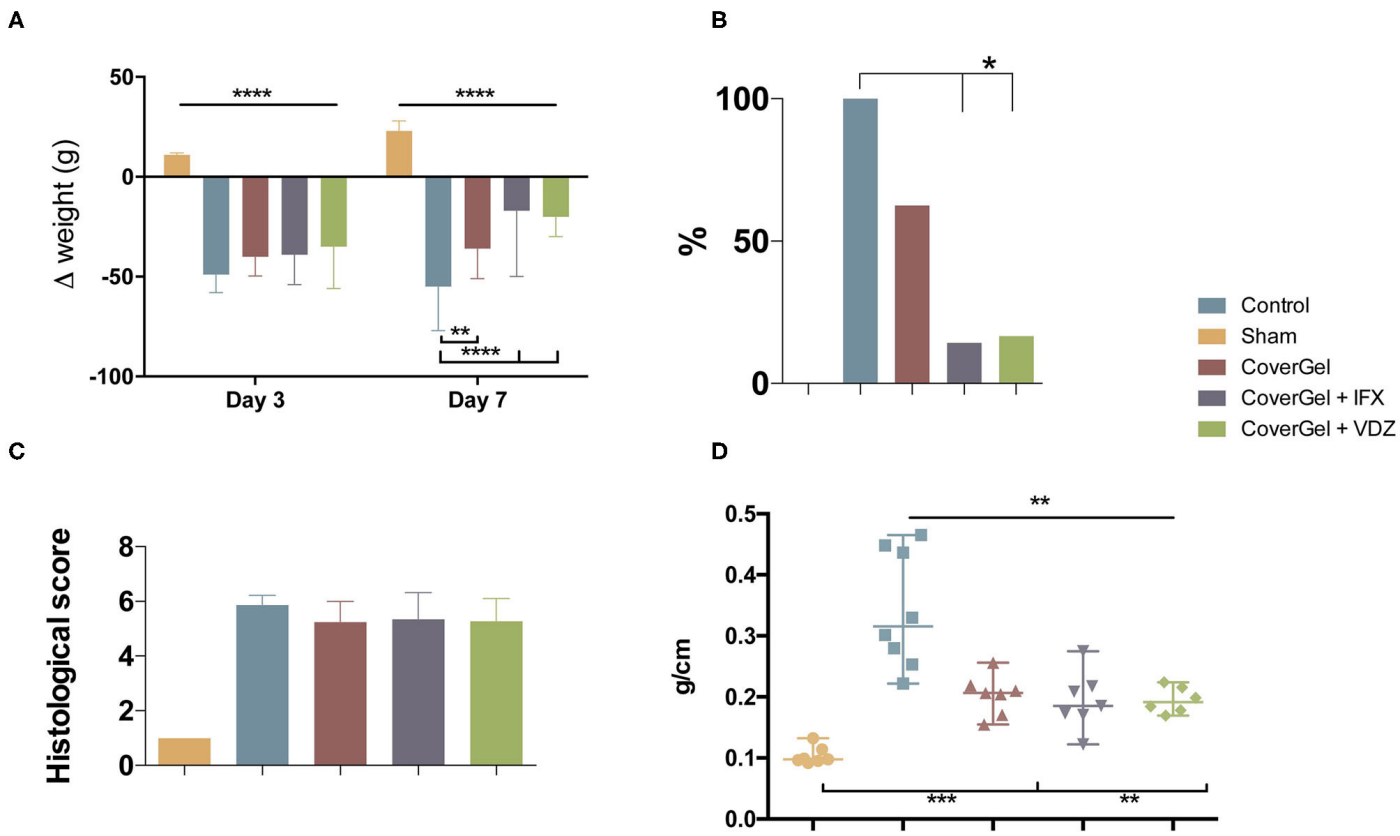
Clinical evaluations on rats from the acute EC animal model. Color representations are as follows: 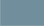 Control group 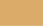 Sham group 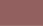 CoverGel group 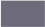 CoverGel + IFX group and 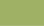 CoverGel + VDZ group. **(A)** Variation (Δ) of weight relative to day 0 (day of induction. **(B)** Bacterial translocation to the liver. **(C)** Histological score. **(D)** Colon weight/length ratio. *p < 0.05; **p < 0.01; ***p < 0.005; ****p < 0.001.

All animals with acute EC had lost more than 8% of original weight at day 3. At day 7, control animals showed a significant weight loss (−55%) as compared with the other groups. The drug-eluting platform with IFX or VDZ showed a significant weight recovery in comparison with CoverGel without bioactive drug (−17 and −20 vs. −36%; *p* < 0.044) (Figure 6A). BT to liver at day 7 was absent in sham animals. The Control group presented a significantly higher BT as compared with CoverGel + IFX and CoverGel + VDZ (100 vs. 14.3 and 16.6%; *p* < 0.05). Animals treated with CoverGel showed a decreased BT (62.5%) without statistical differences (Figure 6B). The histological score for groups with EC was comparable: Control 5.87 ± 0.35, CoverGel 5.24 ± 0.76, CoverGel + IFX 5.34 ± 0.98, and CoverGel + VDZ 5.28 ± 0.82 (Figure 6C). The ratio between colon length and weight [median (range)], a simple indicator of edema and inflammation in the tissue, was significantly higher in control animals as compared with the other treatment groups (Control vs. CoverGel, CoverGel + IFX and CoverGel + VDZ) [0.3157 (0.244) g/cm vs. 0.2063 (0.1012) g/cm *p* = 0.0012, 0.1854 (0.1527) g/cm *p* = 0.0012 and 0.1914 (0.0545) g/cm *p* = 0.0013], respectively (Figure 6D).

### Chronic EC Animal Study

**Figure 7:**
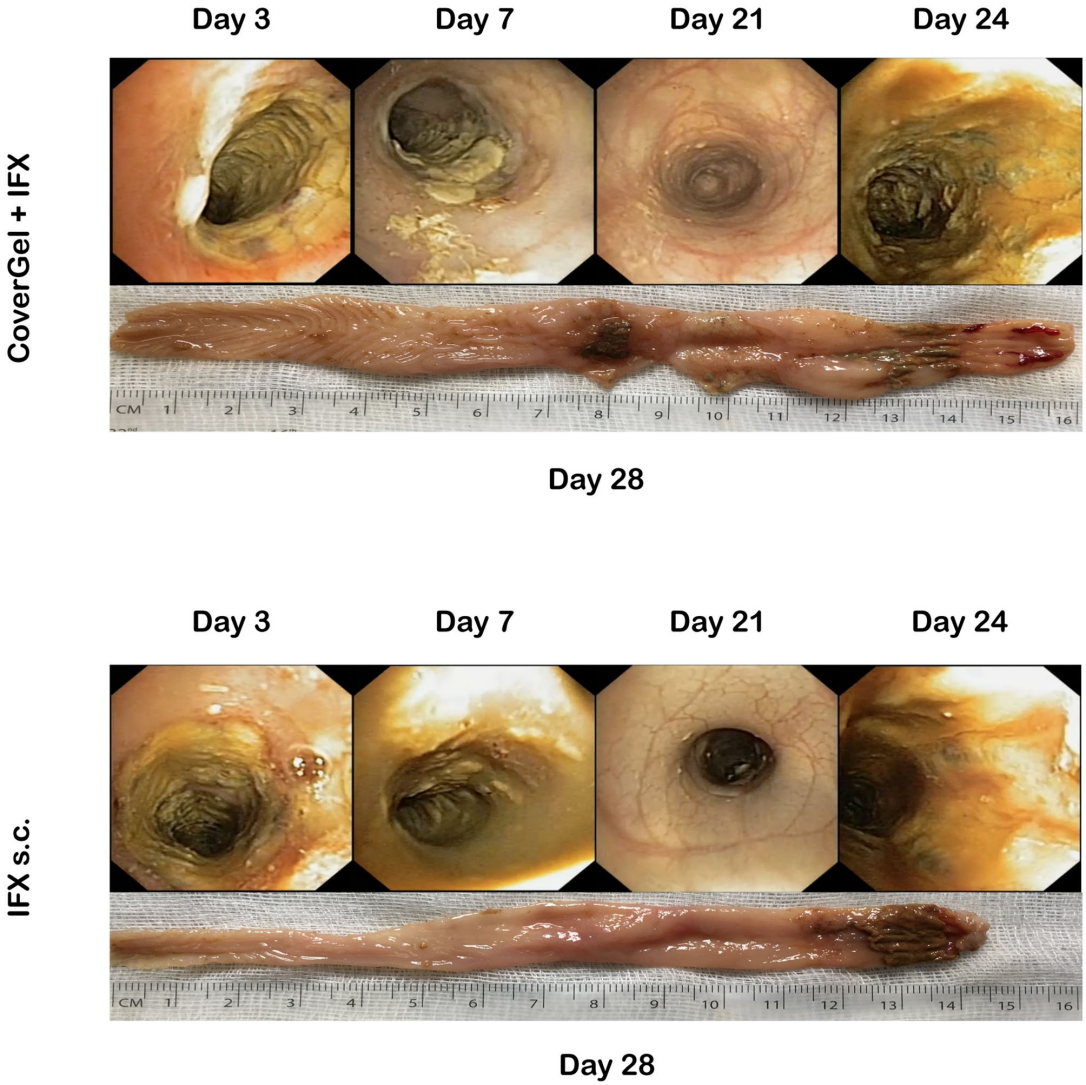
Endoscopic and macroscopic representative images of the chronic EC animal model. Endoscopic images from day 3 (first treatment application), day 7 (second TNBS induction), day 21 (fourth TNBS induction), day 24 (fourth treatment application), and macroscopic image of day 28 (day of euthanasia).

There was variation in both groups in the degree of colitis induction throughout the 4 TNBS applications. Figure 7 shows representative endoscopic and macroscopic images of both groups.

**Figure 8:**
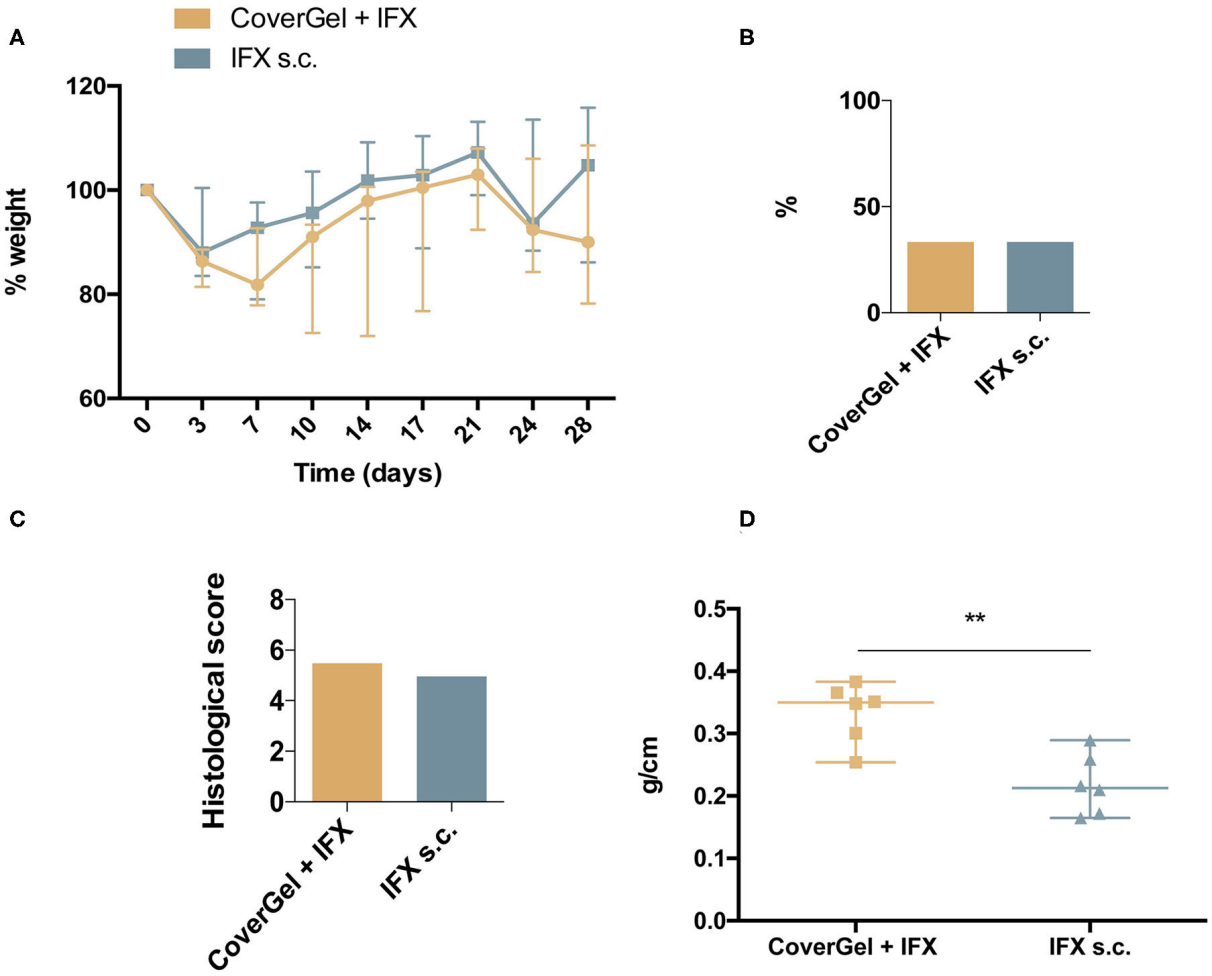
Clinical evaluations on rats from the acute EC animal model. Color representations are as follow: 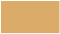 CoverGel + IFX group and 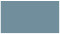 IFX s.c. group. **(A)** Variation (Δ) of weight relative to day 0 (day of induction). **(B)** Bacterial translocation to the liver. **(C)** Histological score. **(D)** Colon weight/length ratio. **p < 0.01.

No statistically significant differences were observed in ponderal evolution at any day of the study (Figure 8A), in BT to liver (two animals out of six in each group, 33.3%) (Figure 8B), and in the histological score for CoverGel + IFX (5.48 ± 0.55) and IFX s.c. (4.96 ± 0.71) (Figure 8C). Colon weight/length ratio at day 28 was significantly lower in the IFX subcutaneous treated group compared to the group treated with CoverGel + IFX (0.213 (0.124) vs. 0.3497 (0.129), *p* = 0.0087) (Figure 8D).

**Figure 9:**
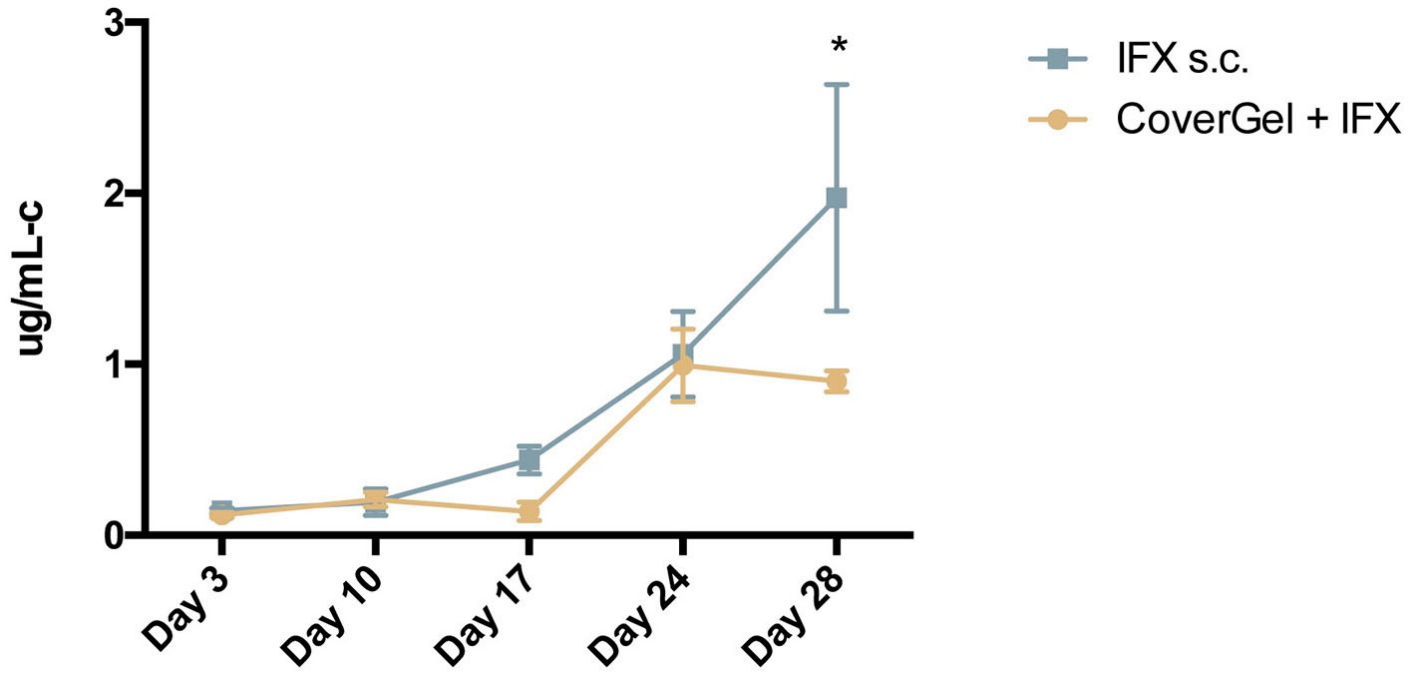
Evolution of antibodies to Infliximab (ATIs) in serum for: 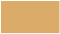 CoverGel + IFX group and 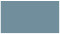 IFX s.c. group. Graph represents the concentration of ATIs (μg/mL-c calibrated in comparison to a polyclonal rabbit α-IgG standard). *p < 0.05.

The evolution of levels of ATIs for each group is shown in Figure 9. At day of euthanasia, levels of ATIs were significantly lower in rats treated with CoverGel + IFX compared to rats treated with IFX subcutaneously (0.90 ± 0.06 vs. 1.97 ± 0.66 μg/mL-c, *p* = 0.0025).

## Discussion

In this paper we have reported the development and characterization of a new hydrogel, the feasibility of the application of this hydrogel as a drug-eluting platform, and the effectiveness of this platform to treat local inflammatory lesions in the colon. This new hydrogel is based on Poloxamer Pluronic-F127, Hyaluronic acid, and Methylcellulose which present biocompatible, bioadhesive, and bioactive properties. Alone, these three components of which our hydrogel is composed have proven their biocompatibility among other beneficial characteristics (10–14). HA is well-known for its promotion of cellular viability which is thought to be via the CD44 receptor and the IL6 pathway; this effect has also been seen in tumor cells (15–17). This could represent a problem since an inflammatory environment such as a UC increase the risk of tumor development. However, despite this, HA hydrogels loaded with antitumoral drugs have been very useful in inducing cancer cell death (18) in truly tumor environments, so we assumed that this cellular viability enhancement helps in the mucosal restoration but would not be enough to cause tumor development.

Mucosal lesions refractory to biological treatments in IBD patients is a clinical challenge and unmet need that requires new treatment modalities. Drug delivery directly to the colon is an option, thanks to oral dosage formulations that are designed to achieve a controlled drug release. However, this controlled release has mainly been achieved with small molecules but not for macromolecules, such as biologics (10). Direct release in the colon is beneficial to prevent early absorption of the drug, thus increasing the quantity of drug reaching the target tissue and at the same time decreasing systemic exposure and associated side-effects. A specific form of control drug release is polymer-based drug delivery. Polymers offer a wide range of possibilities useful for colonic delivery. These polymers can be triggered to start releasing drug by physiological characteristics of the healthy colon, as well as in different disease situations (10). Hydrogels for drug delivery have also been studied for their capacity of mucoadhesion and controlled delivery (11).

Local injection of infliximab can be a novel scenario for endoscopic therapy in IBD patients with symptomatic isolated mucosal lesions, as has been proposed by our group (12). The real efficacy of locally injected IFX is not discernible from current literature, but available data seem encouraging at a dose of 100 mg per session, assuming that higher anti-TNF concentrations given through local injection can favor healing and prevent complications, because a trend for a dose–response curve was observed. Topical injection of anti-TNF agents has been described in the treatment of long-standing perianal fistulizing CD in the absence of an abscess (2). There are technical variations in topical injection of the anti-TNF agents. The agents were injected in a circumferential or intrafistula fashion via the external or internal.

In this work, we have provided a new therapeutic tool for endoscopy by developing a new hydrogel (CoverGel) based on HA, MC, and F127, which presents biocompatible, bioadhesive, and bioactive properties (13–17). CoverGel is an endoscopic vehicle to locally deliver biological drugs (IFX and VDZ) with proven efficacy in acute and chronic EC in rats. Moreover, the endoscopic administration of IFX induces significantly lower levels of ATI as compared with subcutaneous administration. The rationale for the drug dosages used (1 mg/mL) with our drug-eluting gel is based on previous clinical experience with local injection of IFX (12).

Our developed hydrogel showed a reverse thermosensitive capacity of gelation accompanied by a greater adhesion capacity. When the hydrogel contacts the mucosal layer at a physiological temperature, it gelifies permitting the adhesion to the mucosa (18). Rheological studies in our hydrogel confirmed that it is a non-Newtonian fluid, meaning that its viscosity varies with temperature. Morphologically, our hydrogel presents a macroporous (pore diameters > 50 nm) interconnected network that can be the matrix for cell ingrowth as well as nutrients and waste flow—essential characteristics for cell survival within the hydrogel. This platform is less vulnerable to matrix transformations due to osmotic pressure or tissue extrusion, which is an advantage for a controlled drug release of the hydrogel (19). Different kinetic models were studied with the results obtained from the drug release experiments. The kinetic model that best fitted our results was the Higuchi release model. This model is explained by Fick's law, in which the diffusion of a molecule is related to its concentration. The diffusion flux goes from a higher concentrated area to a lesser concentrated one. The magnitude of the diffusion flux will be proportional to the concentration gradient. These results and kinetic model were confirmed when mechanistic of drug release from Covergel were studied by the Korsmeyer-Peppas model, confirming that the diffusion coefficient follows a Fickian diffusion. Our hydrogel showed the capacity to improve cellular viability in a dose-dependent manner at least until a 10%, demonstrating a good potential to become a cell carrier. Intraperitoneal injection stated that CoverGel did not cause an acute toxicity reaction on animals 3 or 7 days post intraperitoneal injection. Subcutaneous placement of our hydrogel in rats was done to evaluate *in vivo* behavior and biodegradability of our hydrogel, which was evaluated by SEM, and we confirmed that the inner structure was maintained 10 days after s.c. placement and no cell infiltration was seen.

All of these results help us conclude that this hydrogel is a good candidate to be used in the digestive system and implement in endoscopic techniques in order to cover some unmet needs of the field. Although *in vitro* and *in vivo* results are really promising, further experiments on *in vivo* drug release kinetics and degradation need to be performed, as these characteristics have previously been reported to vary from *in vitro* analysis to *in vivo*, due to inflammatory cell response (20–22).

TNBS-induced colitis has been proven as a valid method to elucidate different mechanisms underlying the mechanism of the disease (23). Furthermore, and besides its limitations mimicking all different aspects of this complex disease, which are still unknown, this model is a great tool to study the immunopathogenesis of the disease and the potential benefit of different therapies (23).

Our results show that the simple application of our hydrogel as a dressing on the inflamed tissue by means of a shield improves the overall outcome and restoration of the mucosal layer. This beneficial effect of CoverGel is based on its good biocompatibility properties, able to improve mucosal healing. However, when CoverGel is loaded with biological drugs with proven efficacy treating this disease, the effect is even more pronounced. In this sense, endoscopic response was obtained after 4 days of treatment in all animals treated with CoverGel, but unfortunately, histological remission was not achieved at this time. Despite that we do not have measurements of local and systemic levels of the drugs applied by the hydrogel, clinical response was accompanied by different clinical aspects such as body weight loss, colon weight/length ratio, or bacterial translocation to the liver; all features improved in the groups treated with CoverGel alone or in combination with drugs.

The efficacy of an enema based on ascorbyl palmitate hydrogel loaded with dexamethasone has been demonstrated in mice with colitis as well as in the *ex vivo* analysis of colon samples from patients with IBD (24). By contrast, our hydrogel, which can be also administered as enema, has been designed to be administered under an endoscopic view to perform a more localized treatment. Another goal of our project was to improve the patient developing resistance to anti-TNF agents, which is one of the main problems associated with biologics. This resistance is caused by the formation of antidrug antibodies (ADAs), as a response of the body to these exogenous complex proteins. The kind of mAbs most used in IBD, no matter their nature, cause to a greater or lesser degree ADA formation (25). Since new drug development is a complex and long-lasting effort, new therapeutic approaches are needed to reduce this risk and allow patients to continue on a successful therapy longer. The direct drug administration with CoverGel was able to significantly improve the disease outcome in an animal model using a much lower dose than the standard dose applied. Moreover, when we compared the formation of ATIs from subcutaneous injection vs. CoverGel application in an experimental model of chronic EC (with a longer period of time of the disease state), we observed that whereas there were no major significant differences on the outcome of the disease between both ways of treatment application, the levels of ATIs were significantly lower on the group treated with CoverGel. These results confirmed our theory that a locally applied therapy at a lower dose is able to obtain a good clinical result and at the same time reduce adverse events and complications associated with the systemic application of a drug.

Taken together, the results from our study, although preliminary and preclinical, demonstrate that endoscopic administration of a based-on hydrogel drug delivery platform represents a safe and promising approach for the management of active lesions with a lower dose as compared with systemic administration. Present findings also suggest than levels of ADAs are lower with this approach, opening the opportunity to be used in patients with a past history of acute infusion reactions to the drug. Further research, preclinical and clinical, needs to be pursued to safely evaluate and introduce this technique in standard practice to increase the pool of therapeutic interventions to treat IBD patients. Direct drug administration through endoscopy opens new possibilities for therapeutic endoscopy, allowing for the application of bioactive treatments in different pathologies already managed with endoscopy such as colorectal cancer and local inflammatory lesions.

## Data Availability Statement

All datasets generated for this study are included in the article/supplementary material.

## Ethics Statement

The animal study was reviewed and approved by Animal Experimentation Ethics Committee of the Hospital Universitari Germans Trias I Pujol (registered as B9900005).

## Author Contributions

IB, RB, and VL-Z conceived and designed the experiments. IB, MC-S, RB, and VL-Z performed the experiments. IB and NO analyzed the data. RB, VL-Z, and MC-S contributed reagents, materials, and analysis tools. IB, RB, and VL-Z wrote the paper. All authors contributed to the article and approved the submitted version.

## Conflict of Interest

VL-Z and RB are coauthors of a patent for the “BIOADHESIVE PLATFORM TO PERFORM BIOACTIVE TREATMENT” International application number “PCT/EP2017/068876” and International Publication Number “WO 2018/019881 A1” placed in the WIPO/PCT (World Intellectual Property Organization/Patent Cooperation Treaty). The remaining authors declare that the research was conducted in the absence of any commercial or financial relationships that could be construed as a potential conflict of interest.
