# Corrigendum: Development and Characterization of a New Endoscopic Drug-Eluting Platform With Proven Efficacy in Acute and Chronic Experimental Colitis

**DOI:** 10.3389/fmed.2021.785148

**Published:** 2021-10-26

**Authors:** Ignacio Bon, Mary Cano-Sarabia, Napoleon de la Ossa, Ramon Bartolí, Vicente Lorenzo-Zúñiga

**Affiliations:** ^1^Health Research Institute Germans Trias i Pujol (IGTP), Barcelona, Spain; ^2^Catalan Institute of Nanoscience and Nanotechnology (ICN2), CSIC and The Barcelona Institute of Science and Technology, Campus de la UAB, Bellaterra, Spain; ^3^Servicio de Anatomia Patológica, Hospital Universitari General de Catalunya—Grupo Quirón Salud, Barcelona, Spain; ^4^Centro de Investigación Biomédica en Red de Enfermedades Hepáticas y Digestivas (CIBEREHD), Barcelona, Spain; ^5^Endoscopy Unit, University Hospital La Fe, Valencia, Spain

**Keywords:** drug-eluting, experimental colitis, endoscopic shielding technique, hydrogel, endoscopy

There is an error in the **Funding** statement. Instead of “Health Institute carlos III”, the correct name for the funder is “Instituto de Salud Carlos III (Co-funded by European Regional Development Fund/European Social Fund “A way to make Europe”/“Investing in your future”)”.

The authors apologize for this error and state that this does not change the scientific conclusions of the article in any way. The original article has been updated.

## Publisher's Note

All claims expressed in this article are solely those of the authors and do not necessarily represent those of their affiliated organizations, or those of the publisher, the editors and the reviewers. Any product that may be evaluated in this article, or claim that may be made by its manufacturer, is not guaranteed or endorsed by the publisher.

